# Cardiovascular Regeneration: Biology and Therapy

**DOI:** 10.1155/2017/4163694

**Published:** 2017-04-09

**Authors:** Changwon Park, Kiwon Ban, Rebecca D. Levit, Wenbin Liang, Hun-Jun Park, Mary B. Wagner

**Affiliations:** ^1^Department of Pediatrics, Emory University School of Medicine, 2015 Uppergate Dr, Atlanta, GA 30322, USA; ^2^Department of Biomedical Sciences, City University of Hong Kong, 1B-205, 2/F, Block 1, To Yuen Building, Kowloon Tong 999077, Hong Kong; ^3^Division of Cardiology, Department of Medicine, Emory University School of Medicine, 101 Woodruff Circle, Atlanta, GA 30322, USA; ^4^University of Ottawa Heart Institute and Department of Cellular and Molecular Medicine, University of Ottawa, 40 Ruskin St., Ottawa, ON, Canada K1Y 4W7; ^5^College of Medicine, Cardiovascular Center, Seoul St. Mary's Hospital, The Catholic University of Korea, 222 Banpodaero, Seocho-ku, Seoul 137-040, Republic of Korea

Despite a multitude of advancements and efforts, morbidity and mortality rates of patients with ischemic cardiovascular diseases (CVDs) remain high. Recent progress in the fields of cardiovascular biology and stem cell and tissue engineering not only provides a detailed insight into the cardiovascular pathology but also opens opportunities to develop novel therapeutic approaches. Accordingly, this special issue provides an in-depth and up-to-date insight into cardiac regeneration covering key signaling pathways, stem cell-based or cell-free therapeutic strategies and generation of human cardiac myocytes for cell therapy.

Fueled by a rapid development of experimental techniques, stem cells such as embryonic stem cells, induced pluripotent stem cells, and adult stem cells have gained an extensive attention. In the article “Therapeutic Potential of Stem Cells Strategy for Cardiovascular Diseases” by C. Y. Lee et al., the authors thoroughly documented the efficacy and safety of stem cells and stem cell-derived extracellular vesicles for the treatment of CVDs. Also, they briefly touched limitations of these current approaches.

The review article by T. Simard et al. specifically discussed the latest information of endothelial progenitor cells (EPCs), which have been considered as one of the most promising cell sources for arterial repair. The authors described the details of the current definitions of EPCs, their sources, and the suggested underlying mechanisms of EPC-mediated vascular repair. Finally, they discussed the possibility of the use of EPCs as therapeutic options, focusing on endogenous augmentation and transplantation. Improved understanding of the fundamental biology of EPCs, which are well described in this article, will advance the use of EPCs as a valuable therapeutic option for vascular repair.

One of the most challenges of cell therapy for CVDs is to obtain cardiomyocytes, which can function to improve or cure the diseases without unexpected detrimental effects upon transplantation. In the article “De Novo Human Cardiac Myocytes for Medical Research: Promises and Challenges” by V. Hamel et al., the authors comprehensively reviewed the latest advances in the generation of human cardiac myocytes from pluripotent stem cells or by direct reprogramming. Due to the species difference, animal study data cannot be directly translated to human. Thus, human cardiac myocytes are of utmost importance for scientific research. This article also discusses the difficulties of using de novo human cardiac myocytes, such as their immature phenotype and heterogeneity. Moreover, advancements in addressing these challenges are described in this article.

Further, L. Wang and colleagues raised a fundamental question regarding a significantly impaired capability of cardiac regeneration in mammalian and discussed important players that can contribute to therapeutic cardiac repair in the article entitled “Repair Injured Heart by Regulating Cardiac Regenerative Signals.” The article covered functional significance of key extracellular, intracellular signals and cardiac transcription factors.

The authors of the articles published in this special issue have discussed the current status of cardiovascular diseases and related research with particular emphasis on therapeutic strategies for cardiac regeneration. Combined and continued endeavor between basic, translational, and clinical research will generate an innovative and efficient means of treating or curing the devastating disease.

*Changwon Park* *Kiwon Ban* *Rebecca D. Levit* *Wenbin Liang* *Hun-Jun Park* *Mary B. Wagner*

